# Supplementary material for: Phylogenomic synteny network analyses reveal ancestral transpositions of auxin response factor genes in plants
Source: Plant Methods. 2020 May 14;16:70. doi: 10.1186/s13007-020-00609-1 (PMC7226935; doi:10.1186/s13007-020-00609-1)
Supplement: Supplementary file 1 — Additional file 1: Fig. S1. Plant lineages screened for ARF homologues. Fig. S2. Number of Auxin Response Factor genes identified from each of the plant genomes. Fig. S3. Terminal branch length comparison between angiosperm and gymnosperm ARF genes. Fig. S4. Phylogenic and synteny network analyses for each of the six groups of ARFs in angiosperms. Table S1. Annotation and classification of ARF genes in Arabidopsis thaliana. [file 13007_2020_609_MOESM1_ESM.docx]

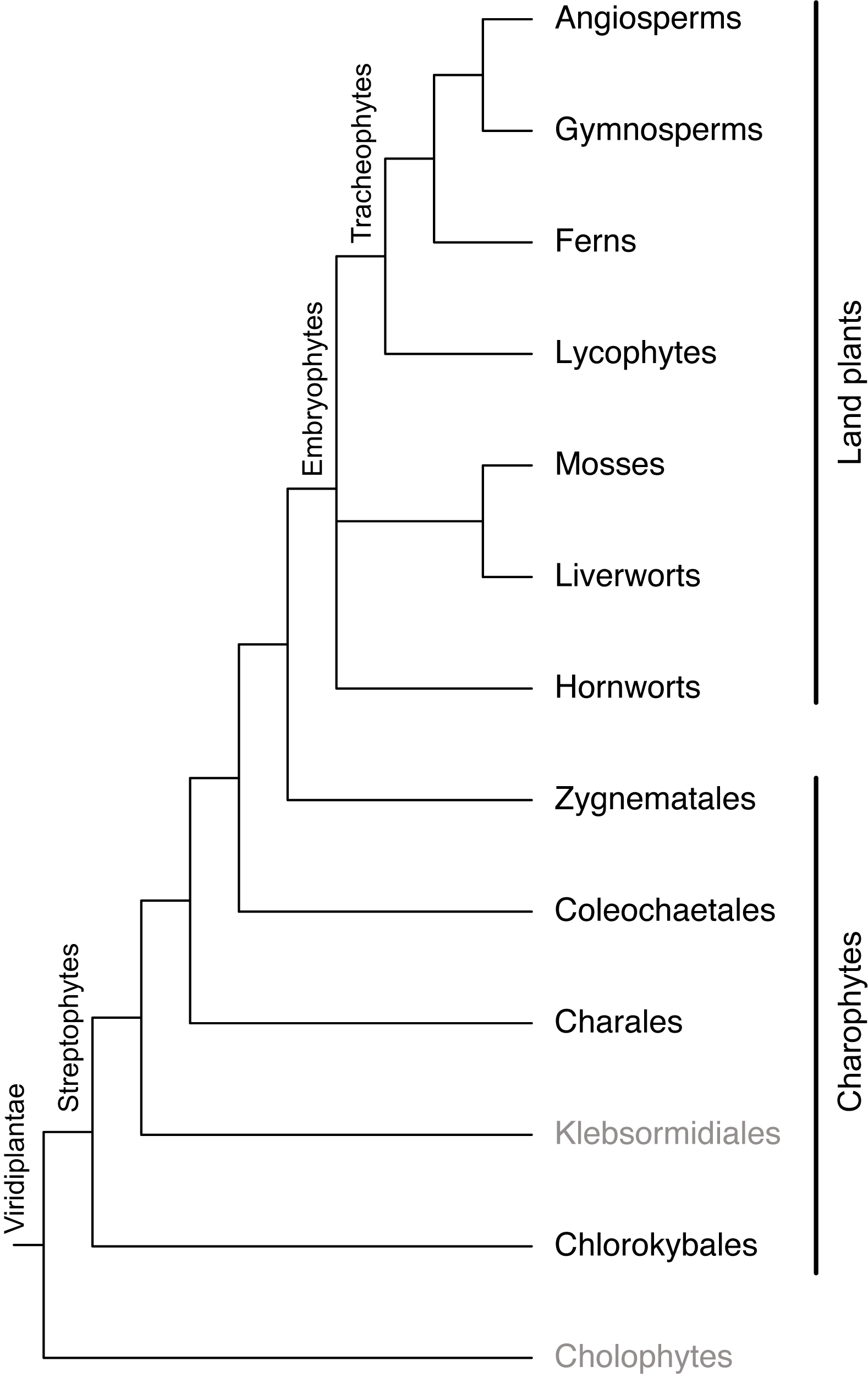


**Fig. S1.** Plant lineages screened for ARF homologues. Auxin response factors were not detected in lineages indicated in grey. Relationships for plant lineages were redrawn from (Wilhelmsson et al., 2017) with minor modifications.


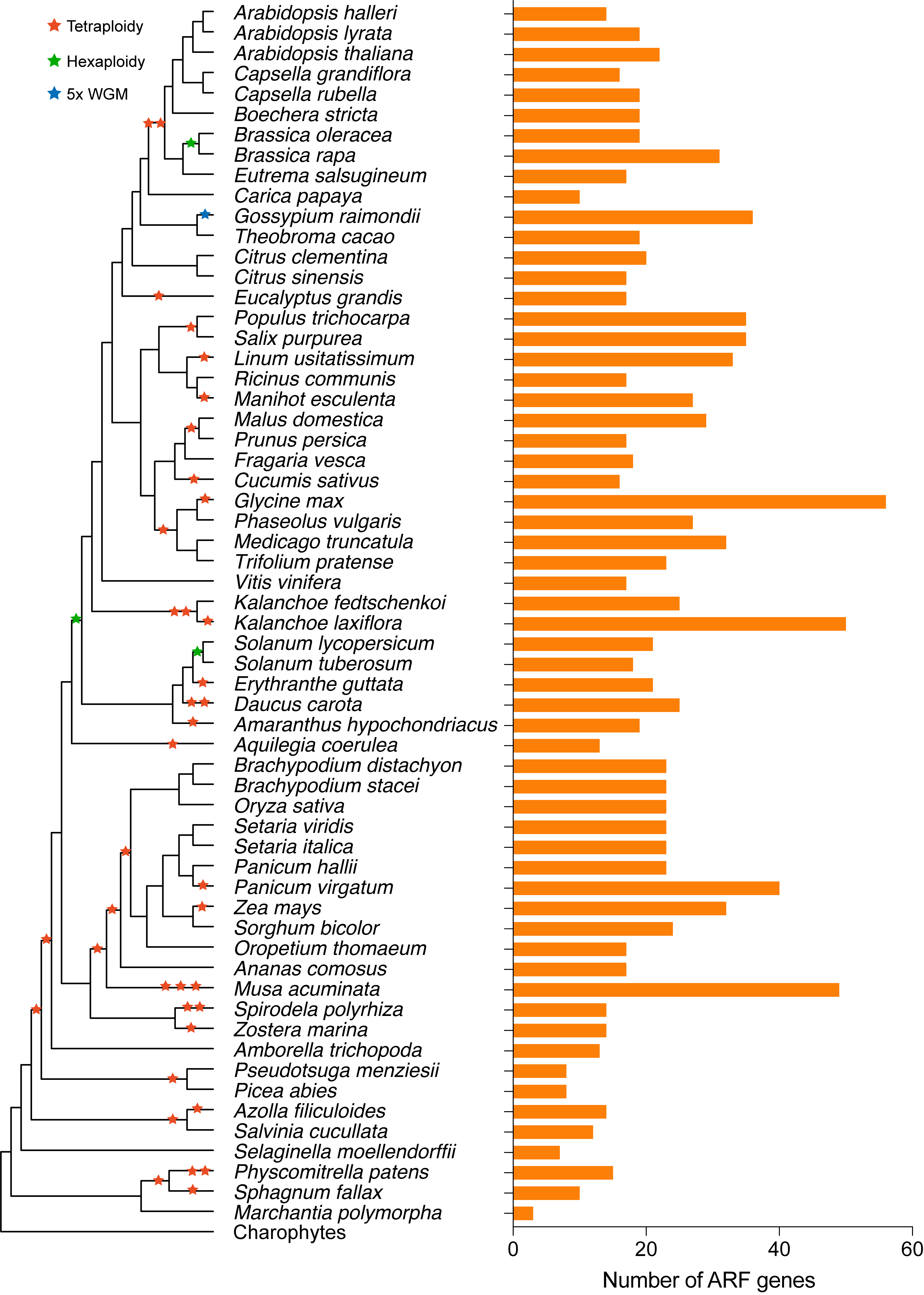


**Fig. S2.** Number of Auxin Response Factor genes identified from each of the plant genomes. The species tree and related paleo-polyploidy events was plotted according to (Van de Peer et al., 2017) with minor modifications.


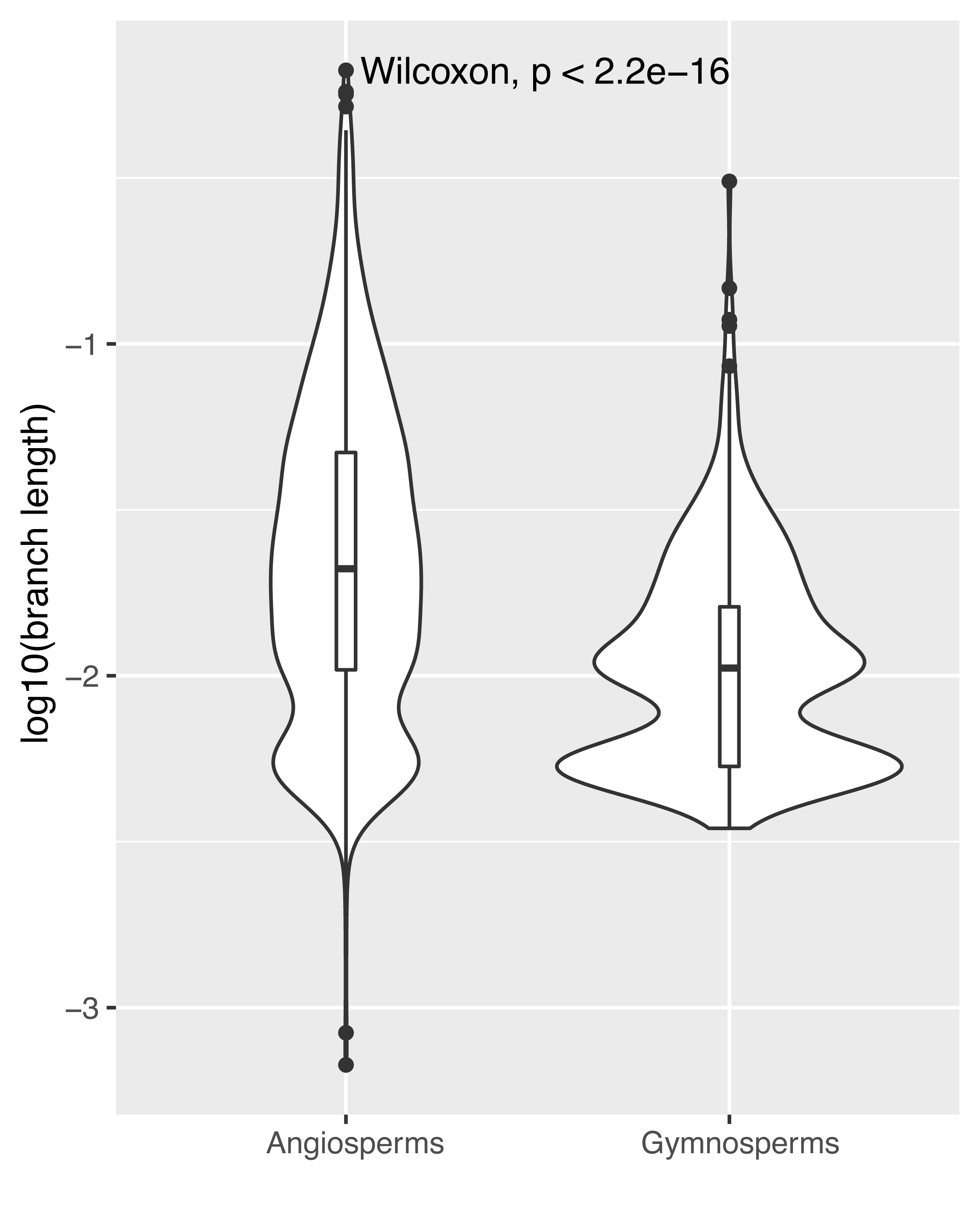


**Fig. S3.** Terminal branch length comparison between angiosperm and gymnosperm ARF genes. Branch lengths estimated in the maximum-likelihood phylogenetic tree of ARF genes were logarithm transformed and compared using a Wilcoxon test.


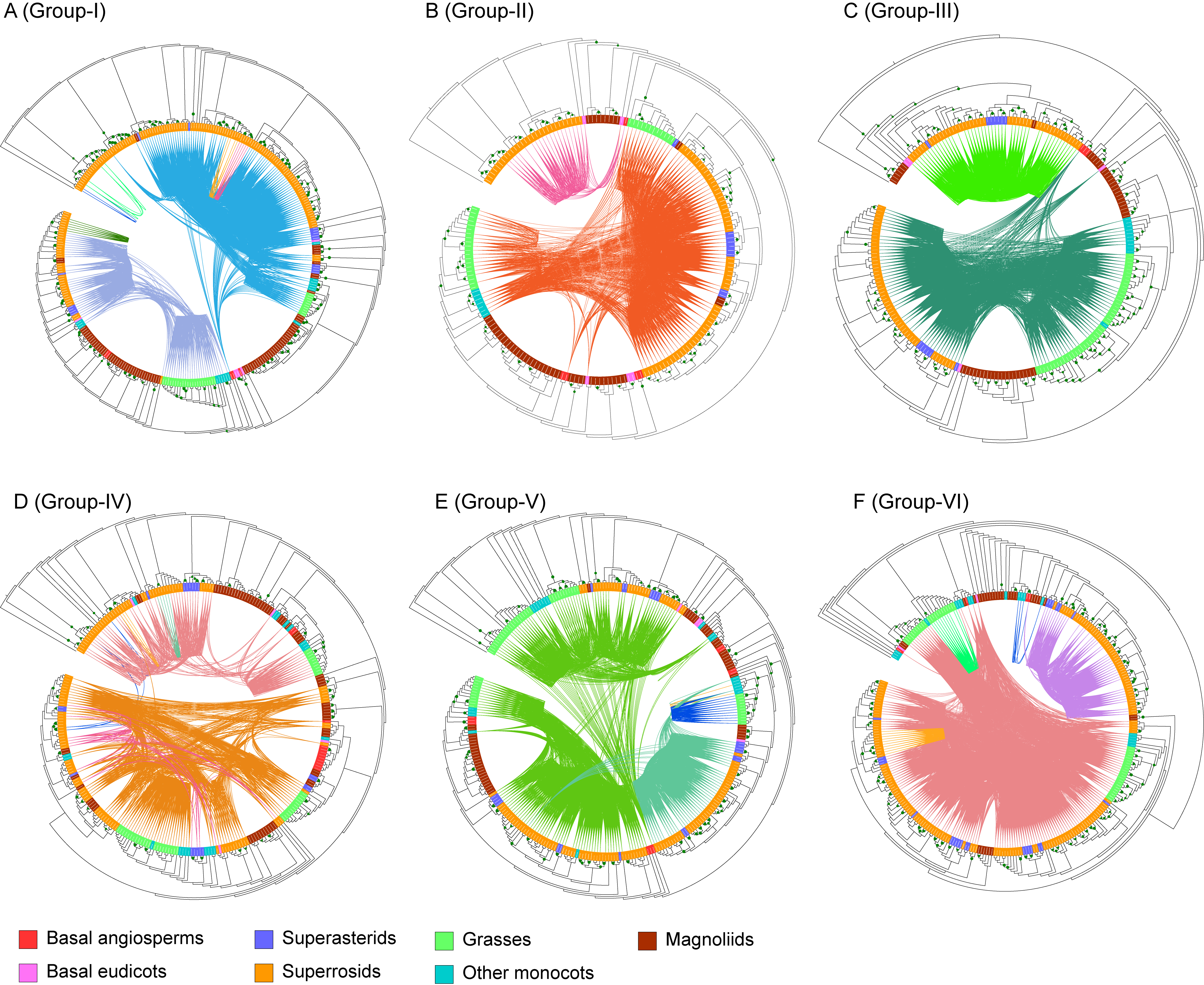


**Fig. S4.** Phylogenic and synteny network analyses for each of the six groups of ARFs in angiosperms. Maximum-likelihood trees (constructed using RAxML) for each of the six ARF groups were constructed, genes from different species groups were colored using different colors and genes detected in syntenic genomic blocks (syntelogs) were connected using curved lines. The syntenic connections belonging to different synteny network communities were plotted using different colors.

**Table S1** – Annotation and Classification of *ARF* genes in *Arabidopsis thaliana*

| Gene ID | TAIR description | Annotation | Finet *et al.* 2013 | This Study |
| --- | --- | --- | --- | --- |
| AT1G34170 | auxin response factor 13 | ARF13 | ARF 9 | Group_I |
| AT1G34310 | auxin response factor 12 | ARF12 | ARF 9 | Group_I |
| AT1G34390 | auxin response factor 22 | ARF22 | ARF 9 | Group_I |
| AT1G34410 | auxin response factor 21 | ARF21 | ARF 9 | Group_I |
| AT1G35240 | auxin response factor 20 | ARF20 | ARF 9 | Group_I |
| AT1G35520 | auxin response factor 15 | ARF15 | ARF 9 | Group_I |
| AT1G35540 | auxin response factor 14 | ARF14 | ARF 9 | Group_I |
| AT1G59750 | auxin response factor 1 | ARF1 | ARF 1 | Group_I |
| AT2G46530 | auxin response factor 11 | ARF11 | ARF 9 | Group_I |
| AT3G61830 | auxin response factor 18 | ARF18 | ARF 9 | Group_I |
| AT4G23980 | auxin response factor 9 | ARF9 | ARF 9 | Group_I |
| AT5G62000 | auxin response factor 2 | ARF2 | ARF 2 | Group_II |
| AT2G33860 | auxin response factor 3 | ARF3 | ARF 3/4 | Group_III |
| AT5G60450 | auxin response factor 4 | ARF4 | ARF 3/4 | Group_III |
| AT1G30330 | auxin response factor 6 | ARF6 | ARF 6/8 | Group_IV |
| AT5G37020 | auxin response factor 8 | ARF8 | ARF 6/8 | Group_IV |
| AT1G19220 | auxin response factor 19 | ARF19 | ARF 5/7 | Group_V |
| AT1G19850 | auxin response factor 5 | ARF5 | ARF 5/7 | Group_V |
| AT5G20730 | auxin response factor 7 | ARF7 | ARF 5/7 | Group_V |
| AT1G77850 | auxin response factor 17 | ARF17 | ARF 10/16/17 | Group_VI |
| AT2G28350 | auxin response factor 10 | ARF10 | ARF 10/16/17 | Group_VI |
| AT4G30080 | auxin response factor 16 | ARF16 | ARF 10/16/17 | Group_VI |
| AT1G43950* | auxin response factor 23 | ARF23 | ARF 9 | Group I |

*The ARF23 in *Arabisopsis thaliana* is a truncated gene.
